# Structure beyond pair correlations: X-ray cross-correlation from colloidal crystals^1^

**DOI:** 10.1107/S1600576716017313

**Published:** 2016-11-08

**Authors:** Felix Lehmkühler, Birgit Fischer, Leonard Müller, Beatrice Ruta, Gerhard Grübel

**Affiliations:** a Deutsches Elektronen-Synchrotron (DESY), Notkestrasse 85, 22607 Hamburg, Germany; b The Hamburg Centre for Ultrafast Imaging, Luruper Chaussee 149, 22761 Hamburg, Germany; c ESRF – The European Synchrotron, 71 avenue des Martyrs, CS 40220, 38043 Grenoble Cedex 9, France

**Keywords:** colloidal crystals, coherent X-ray scattering, small-angle X-ray scattering, X-ray cross-correlation analysis

## Abstract

The results of an X-ray cross-correlation analysis (XCCA) study on hard-sphere colloidal crystals and glasses are presented. The article shows that cross-correlation functions can be used to extract structural information beyond the static structure factor in such systems. In particular, the powder average can be overcome by accessing the crystals’ unit-cell structure. In this case, the results suggest that the crystal is of face-centered cubic type. It is demonstrated that XCCA is a valuable tool for X-ray crystallography, in particular for studies on colloidal systems. These are typically characterized by a rather poor crystalline quality due to size polydispersity and limitations in experimental resolution because of the small *q* values probed. Furthermore, nontrivial correlations are observed that allow a more detailed insight into crystal structures beyond conventional crystallography, especially to extend knowledge in structure formation processes and phase transitions.

## Abstract 2

An X-ray cross-correlation study with emphasis on colloidal crystals is presented and demonstrates how to access higher-order structure beyond pair correlations. In this way symmetries of the crystal can be determined that are inaccessible in conventional crystallography.

## 1. Introduction   

In recent years, cross-correlation analysis of diffraction patterns from coherent X-ray scattering experiments has attracted increasing interest (Wochner *et al.*, 2009 ▸, 2011 ▸). The technique known as X-ray cross-correlation analysis (XCCA) or fluctuation X-ray scattering has been shown to allow access to information on the sample’s structure beyond pair correlations, expressed by the static structure factor 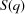. The original proposal of using such correlations goes back to the 1970s, to extract structural information from biological specimens (Kam, 1977 ▸). With the advent of modern synchrotron and free-electron laser (FEL) facilities, the method was for the first time demonstrated experimentally. In these studies, single-particle information has been extracted from cross-correlations of diffraction patterns from ensembles of identical particles (Saldin *et al.*, 2011 ▸; Chen *et al.*, 2012 ▸; Starodub *et al.*, 2012 ▸; Pedrini *et al.*, 2013 ▸).

A different approach using cross-correlations to uncover local structures in amorphous matter was presented first by Wochner *et al.* (2009 ▸). In a coherent X-ray scattering study on densely packed colloidal glass, they found higher-order correlations by XCCA that were related to the local structure of the amorphous sample. This method was further developed by theory and simulations (Altarelli *et al.*, 2010 ▸; Kurta *et al.*, 2012 ▸; Lehmkühler *et al.*, 2014 ▸; Malmerberg *et al.*, 2015 ▸; Latychevskaia *et al.*, 2015 ▸). The first experimental work dealt with correlations in thin colloidal films examined by small-angle scattering (SAXS) (Schroer *et al.*, 2014 ▸, 2015 ▸), the development of bond order in liquid crystals (Kurta *et al.*, 2013 ▸; Zaluzhnyy *et al.*, 2015 ▸) and under pressure (Schroer *et al.*, 2016 ▸), symmetries in magnetic domain patterns (Su *et al.*, 2011 ▸), and the study of correlations in the vicinity of Bragg peaks (Mendez *et al.*, 2014 ▸; Gutt *et al.*, 2014 ▸).

In this study we demonstrate that cross-correlations can be used to extract structural information beyond the static structure factor from three-dimensional colloidal suspensions. In particular, the powder average can be overcome by accessing the unit-cell structure of a colloidal crystal. For a suspension of hard-sphere crystallites, XCCA allows us to access the symmetry of the Bragg reflections, showing that the sample crystallized in a face-centered cubic (f.c.c.) structure. By correlating different Bragg peaks, more details of the colloidal crystal structure can be obtained. This indicates that cross-correlations are a valuable tool to study, for example, the structures of polycrystalline samples or structure formation due to phase transitions.

## 2. Experimental details   

### 2.1. Sample preparation   

We used sterically stabilized poly(methyl methacrylate) (PMMA) hard-sphere particles dissolved in decalin. The PMMA particles were synthesized following previous recipes (Antl *et al.*, 1986 ▸; Pathmamanoharan *et al.*, 1989 ▸) and are known to be a good experimental realization for hard spheres. The spheres had a radius of 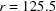
 nm at a polydispersity of 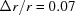, which allows the sample to crystallize. The volume fraction of the crystalline sample was set to 0.52, which corresponds to the crystal–fluid coexistence phase of hard spheres of the given polydispersity (Zaccarelli *et al.*, 2009 ▸; Sollich & Wilding, 2010 ▸). In addition, we studied a glassy sample with a volume fraction of about 0.6. The samples were filled into quartz capillaries with a diameter of 0.7 mm and sealed afterwards. After several months of waiting time, various crystals formed, which were supposed to have f.c.c. structure (Yang & Ma, 2008 ▸; Dolbnya *et al.*, 2005 ▸).

### 2.2. Experimental procedure   

The coherent X-ray scattering experiments were performed at beamline ID10 at ESRF (Grenoble, France). The sample capillaries were placed in the standard sample environment for coherent SAXS experiments. An 8 keV partial coherent X-ray beam with a size of 10 × 10 µm was used, resulting in approximately 10^7^ spheres illuminated by the X-ray beam. The coherent flux was 9 × 10^9^ photons per second. In order to keep the volume fraction constant and neglect influences of sedimentation due to gravity, data were taken in a narrow height section from the capillary. To extract the static structure factor 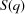 from the scattering patterns, the particle form factor was measured from a diluted sample. The scattering patterns were recorded with a Maxipix 2 × 2 detector with a pixel size of 55 × 55 µm placed approximately 5 m downstream from the sample, resulting in a maximum achievable wavevector transfer of 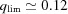
 nm^−1^. The XCCA measurements were performed by taking scattering patterns at 1200 different spots to detect different structural realizations of the sample. In order to achieve reasonable statistics and to check for radiation damage, 10–20 scattering patterns were taken at each sample spot.

## 3. Results   

In XCCA studies, correlations of the intensity are studied by the correlation function (Wochner *et al.*, 2009 ▸; Altarelli *et al.*, 2010 ▸),
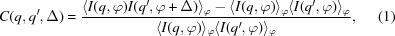
between two wavevector transfers 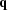, 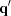 with moduli 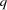, 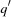. Here, φ denotes the azimuthal angle and Δ the angular difference between the two correlated intensities. Equation (1) is also used in reconstruction studies based on cross-correlations (Pedrini *et al.*, 2013 ▸; Starodub *et al.*, 2012 ▸). For the case of a constant modulus, 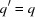, *e.g.* rings of constant wavevector transfer *q* from two-dimensional scattering patterns, the frequently studied case of *q* autocorrelations,
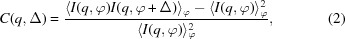
is obtained.

For a more quantitative measure, Fourier coefficients of *C* are studied (Altarelli *et al.*, 2010 ▸; Kurta *et al.*, 2012 ▸). Ensemble averages are necessary to gain access to the overall sample structure. Recently, we showed that this is given by the variance of the Fourier coefficients 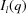 of the intensity 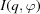 *via* (Lehmkühler *et al.*, 2014 ▸)
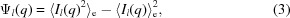
where 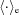 denotes the ensemble average over all realizations, *i.e.* scattering patterns taken from the sample.

### 3.1. Static structure   

**Figure 1:**
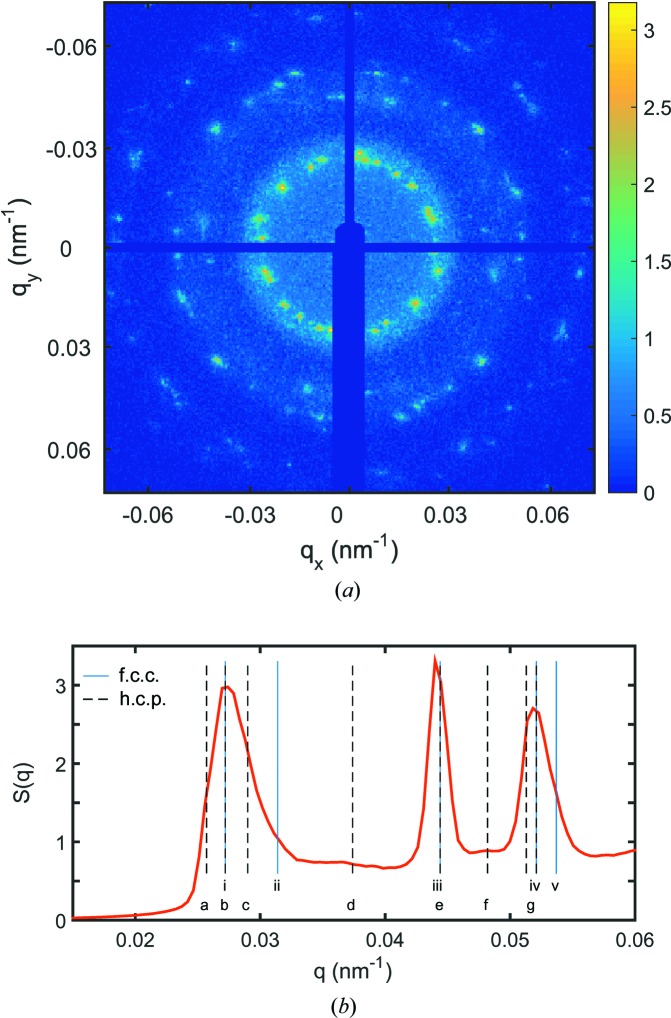
Scattering signal from a crystalline sample. (*a*) Example diffraction pattern. The color bar represents a logarithmic intensity scale. (*b*) Structure factor averaged over all sample spots. The Bragg reflections are indexed for f.c.c. as i 111, ii 200, iii 220, iv 311, v 222, and for the h.c.p. structure as a 010, b 002, c 011, d 012, e 110, f 013, g 020 and 112.

A typical scattering pattern taken from the crystalline sample is shown in Fig. 1 ▸(*a*). Since a finite number of crystals were hit by the beam, a large collection of Bragg reflections is observed, forming very spotted powder rings. In contrast to perfect crystals, for example formed by metal atoms, the study of colloidal crystals with hard X-rays is limited by (1) the comparably poor crystal quality due to shape and size polydispersity and (2) a rather poor *q* resolution in state-of-the-art SAXS experiments compared to conventional crystallographic experiments at large wavevector transfers. Although in principle the resolution may be increased, for example by using analyzer crystals or performing microradian SAXS experiments (Petukhov *et al.*, 2006 ▸), the crystal quality limits the effective resolution. The structure factor averaged over all sample spots is given in Fig. 1 ▸(*b*). Polydisperse hard spheres preferentially crystallize in a random hexagonal close-packed (h.c.p.) structure (Pusey *et al.*, 1989 ▸; Hoogenboom *et al.*, 2003 ▸; Gasser *et al.*, 2001 ▸). However, after sufficient waiting times, such as for our sample, the f.c.c. structure is preferred (Yang & Ma, 2008 ▸). The f.c.c. and h.c.p. reflections are given as lines in Fig. 1 ▸(*b*). Owing to the broad width of the Bragg reflections and limited experimental resolution, several reflections overlap. The analysis of the peak positions results in a lattice constant of 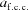 = 400 (5) nm for the case of an f.c.c. structure and consequently 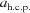 = 283 (4) nm, 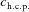 = 462 (6) nm for an h.c.p. structure. This perfectly matches a volume fraction of 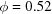. Obviously, both structure models may explain the experimental data, even if not all reflections are well developed, and especially when allowing for small deviations of the lattice constant, for example, due to particle size polydispersity. For a final conclusion on the crystal structure we want to take a closer look at the symmetry of the Bragg peaks by cross-correlation analysis.

### 3.2. Autocorrelations   

**Figure 2:**
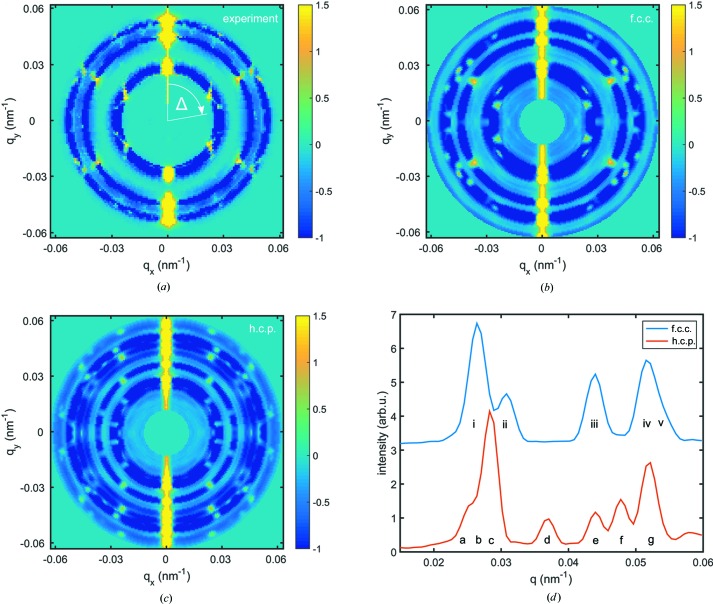
Correlation function 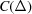 maps for (*a*) the crystalline sample and (*b*) and (*c*) modeled f.c.c. and h.c.p. crystals, respectively. (*d*) Azimuthally averaged intensity for both modeled crystals. The Bragg reflections are indexed in the same way as in Fig. 1 ▸. For comparison, the amplitude of the experimental data is scaled to match the f.c.c. and h.c.p. results given in arbitrary units.

The symmetry of the reflection is not directly accessible, as an unknown but finite number of crystallites with random orientation are hit by the beam. To gain insight into the orientational order, cross-correlation functions 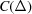 were calculated for *q* = 0.01–0.06 nm^−1^ with a step size of 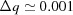
 nm^−1^ (*i.e.* the width of two detector pixels) for every single speckle pattern using equation (2). The raw data were masked for bad pixels, the beam stop shadow and blind pixels at the edge of the four detector elements. Afterwards, the correlation functions were averaged over the ensemble of all 1200 patterns. 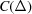 maps taking additional correlations between different *q* values into account have been shown to reflect the diffraction pattern of a single particle in studies on dilute dispersions of equal particles (Pedrini *et al.*, 2013 ▸). In the correlation map shown in Fig. 2 ▸(*a*), 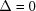 corresponds to the vertical line starting in the center (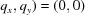 and proceeding upwards (positive 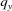) where the dominant speckle autocorrelation is visible. According to Friedel’s law a similar peak is visible at 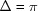 for the case of small scattering angles, which is valid for the data. In the vicinity of the Bragg reflections, correlation peaks can be observed. In this way, the powder average has been overcome, allowing access to different symmetries in the sample.

To compare the sample structure with f.c.c. and h.c.p. crystals, autocorrelation maps have been calculated for such crystals. Perfect crystals were modeled for monodisperse particles of the same size and volume fraction as the sample system in the experiment. Scattering patterns were calculated for 1000 random crystal orientations, and autocorrelations were calculated from each scattering pattern and averaged. The results are shown in Figs. 2 ▸(*b*) and 2 ▸(*c*) for f.c.c. and h.c.p. crystals, respectively. In addition, the azimuthally integrated intensity of each crystal is shown in Fig. 2 ▸(*d*), exhibiting the expected Bragg reflections. Since the resolution of the simulation is higher than that of the experiment, more Bragg peaks can be distinguished (*e.g.* the 111 and 200 reflections of the f.c.c. structure). On comparison with Fig. 1 ▸ it becomes obvious that the experimental resolution and limited quality of the crystals due to the particles’ size polydispersity was not sufficient to draw a final conclusion on the sample’s structure. By comparing the cross-correlation maps on a qualitative level, the experimental data are suggested to better match the f.c.c. map rather than an h.c.p. map; for example, correlations at 0.035 nm^−1^ around the 012 peak of the h.c.p. structure are missing in the experimental data, and the peaks around 0.027 nm^−1^ match the f.c.c. results within the experimental resolution, in contrast to the h.c.p. results. The qualitative agreement is studied by calculation of the Pearson correlation coefficient *R* between the experimental and model maps. We obtain 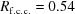 and 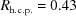, supporting the qualitative observations. Disagreements of the f.c.c. and h.c.p. models may originate from stacking disorder in the crystallites, which might create additional correlation signals and distort the theoretically observed correlation peaks.

**Figure 3:**
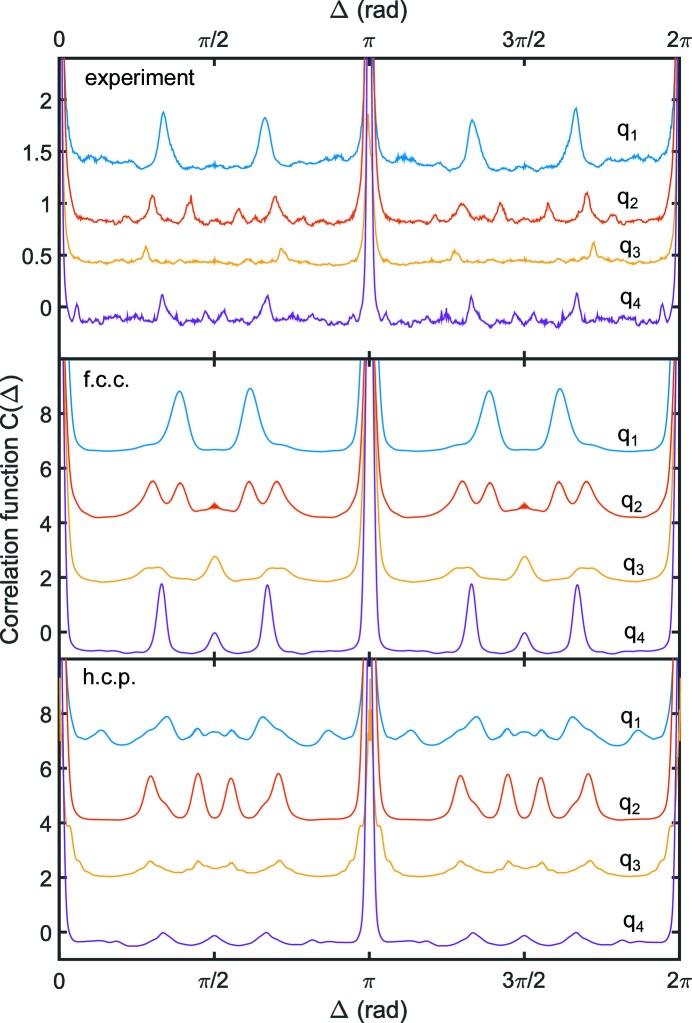
Ensemble averaged cross-correlation functions 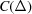 for the experimental data (top), the modeled f.c.c. crystal (middle) and the modeled h.c.p. crystal (bottom). Results are shown for 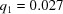
 nm^−1^, 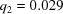
 nm^−1^, 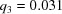
 nm^−1^ and 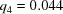
 nm^−1^. The curves are shifted for clarity. Owing to the low signal-to-noise level even very weak correlation peaks can be detected.

Next, we want to expand the observation from the correlation maps and take a closer look at the correlation in the vicinity of the first and second Bragg peaks around 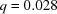
 nm^−1^ and 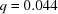
 nm^−1^ (see Fig. 3 ▸). Here, correlations are shown from the experiment and both models at four *q* values (denoted 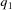
–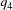). The definitions of the *q* values are given in Table 1 ▸, together with the corresponding Bragg reflections for f.c.c. and h.c.p. crystals. For the experimental data, a well developed sixfold symmetry can be found at 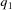 (top), reflecting the typical diffraction signal from a hexagonal plane, as found for example for f.c.c. (111). With increasing *q*, each correlation peak splits into two weaker peaks (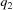). At 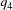, a well developed sixfold order with smaller peaks in the vicinity of 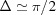 is visible.

First, we compare the experimental results with the findings for the perfect f.c.c. crystal. At 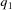, the f.c.c. crystal shows a similar 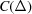, but peaks at slightly different values of Δ. In general, correlations of Bragg peaks appear at angles between equivalent crystal planes; for example, for the 111 reflection of f.c.c., correlations are observed at 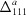 = 70.5° and, owing to the Friedel symmetry of correlations about 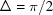 (Kam, 1977 ▸), at 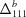 = 180 − 70.5° = 109.5° (Mendez *et al.*, 2014 ▸), in contrast to the multiples of 60° observed in the experimental data as discussed in the previous paragraph. However, the width and shape of the peaks in the experimental data suggest contributions at 70.5 and 109.5° that are superimposed by the sixfold order discussed above. Differences between experiment and the f.c.c. structure can be observed as well at 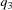. Here, the f.c.c. model shows correlations at 90 and 270° that can be connected to the symmetry of the (200) crystal plane of the f.c.c. structure. In the case of the 200 reflection, correlations are expected for multiples of 90°, such as correlating 200 and 020 reflections. Moreover, broad peaks around 50° and corresponding angles (130, 230 and 280°) are visible. In the experimental data the peaks are rather weak at this *q* value. While the correlations at 50° and corresponding angles are visible, the peaks at 90 and 270° are absent.

In contrast, at 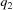 and 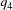, the experimental data and the f.c.c. model agree well: for example, with respect to the correlation peaks at 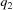. These correlation peaks, in particular at 54°, can be attributed to a correlation of the 111 and 200 or equivalent peaks (Mendez *et al.*, 2014 ▸), where, for example, an angle of 54.7° is expected for 111 and 200 correlations. Owing to the weak effective resolution in SAXS experiments on colloidal crystals as discussed above, close Bragg reflections may fulfill the Laue conditions at the same time owing to the weak resolution, as observed at 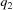. At 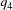, the experimental data resemble the f.c.c. expectations, *i.e.* strong peaks at multiples of 60° and weaker peaks close to 90°. Owing to the 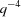 drop of intensity, the results at 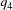 are dominated by the weaker counting statistics and thus the peaks are less developed compared to those calculated for the perfect f.c.c. crystal.

In a second step, we compare the experimental results with the h.c.p. structure. In contrast to the f.c.c. structure, we find less agreement between experiment and the calculated structure. While at some *q* values both f.c.c. and h.c.p. show similar agreements, for example the positions of the correlation peaks at 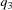 and the same number of correlation peaks at 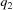, the overall agreement is weaker compared to the f.c.c. structure. In particular, at 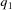 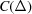 shows a completely different shape. Furthermore, the deviation of the number of peaks is stronger than for the f.c.c. structure (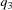) and the (relative) intensities are not well reflected (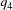). We thus conclude that the experimental results match the f.c.c. results well. We attribute deviations such as weaker peaks, absence of correlation peaks and slight differences in Δ to limited crystal quality, stacking faults typical for polydisperse hard-sphere colloids and limited statistics in the experimental data.

### 3.3. Cross-correlations   

**Figure 4:**
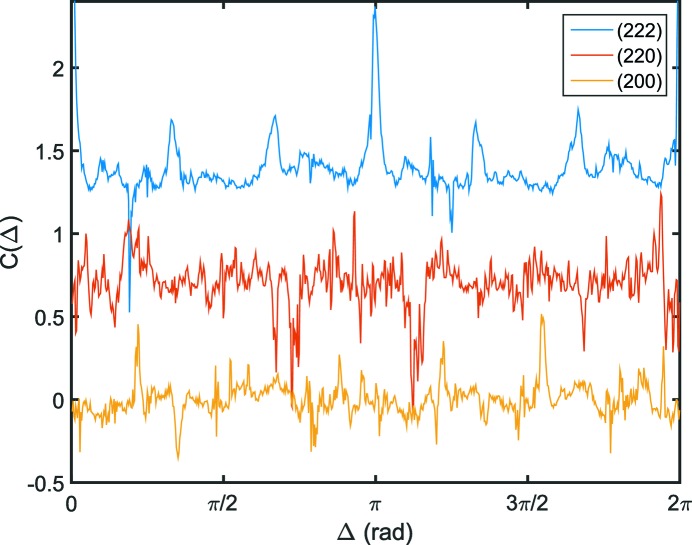
Cross-correlations 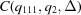 with 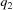 corresponding to the *q* values of the 222, 220 and 200 reflections, respectively. The curves are shifted vertically for clarity.

Here, we focus on correlations with respect to the 111 reflection following equation (1) with 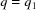. In Fig. 4 ▸ the correlations between the 111 reflection and the 222, 220 and 200 reflections are shown. Not surprisingly, the correlation between 111 and its higher order 222 reflects a sixfold correlation between the two planes. In contrast to the other two planes, the correlations at 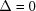 and 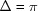 reflecting speckle autocorrelation and Friedel’s law are visible in the case of correlation between the two planes. For the 220 reflection, no correlation to the (111) plane can be observed. In the case of the correlation function between the 111 and 200 reflections, small peaks appear close to the previously discussed angle of 54.7° for 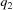. Such correlations indicate simultaneous observation of both Bragg reflections from the same crystal domain. As discussed above, these may originate from the weak resolution and thus simultaneous observation of close Bragg reflections. In addition, such correlation has been observed in correlation studies on atomic length scales and might be connected to orientation of surface facets (Mendez *et al.*, 2014 ▸).

### 3.4. Fourier modes   

**Figure 5:**
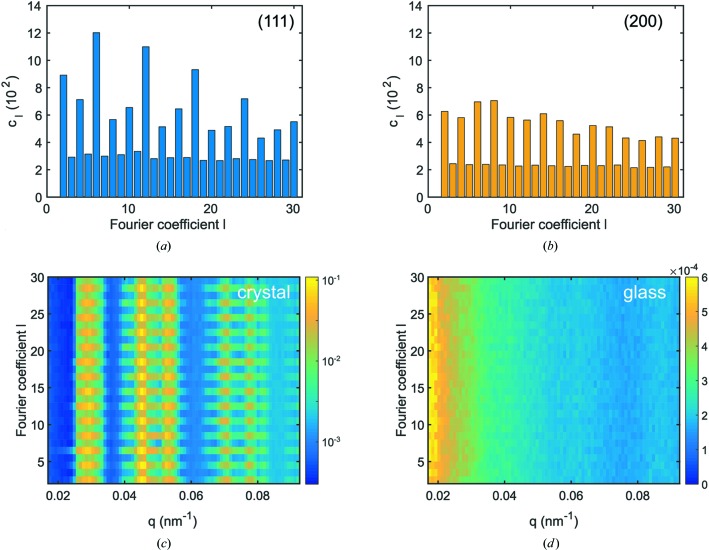
Fourier analysis. (*a*), (*b*) Fourier coefficients 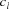 for 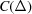 from Fig. 3 ▸ of the 111 and 200 Bragg reflections, respectively. (*c*), (*d*) 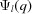 for the crystalline and glass samples, respectively. For clarity, 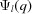 of the crystalline sample is shown in logarithmic scale.

A more detailed insight into the sample structure is typically obtained by a Fourier analysis of the cross-correlation functions, as done in recent XCCA work (Lehmkühler *et al.*, 2014 ▸; Schroer *et al.*, 2014 ▸, 2015 ▸; Zaluzhnyy *et al.*, 2015 ▸; Kurta *et al.*, 2015 ▸). An overview of the analysis is given by Lehmkühler *et al.* (2014 ▸). Because of the symmetry of the diffraction pattern, even Fourier coefficients dominate. Odd coefficients are not equal to zero but finite, which can be connected to non-perfect wavefronts, detector noise and statistics (Lehmkühler *et al.*, 2014 ▸; Schroer *et al.*, 2014 ▸), or more generally, to non-flat Ewald spheres (Liu *et al.*, 2016 ▸). In Figs. 5 ▸(*a*) and 5 ▸(*b*) Fourier modes 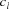 of the 111 and 200 reflections from the crystal sample are illustrated for the averaged correlation function shown in Fig. 3 ▸. The 111 reflection is characterized by strong contributions of sixfold symmetry, *i.e.* (local) maxima for *l* = 6, 12, 18, 24,…, which resemble the sixfold symmetry observed in Fig. 3 ▸. Owing to the small width of the Bragg reflections, higher orders of 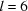 become observable, in particular visible for the averaged correlation function. For the 200 reflection, all even coefficients peak, reflecting the crystal symmetry at 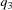. In contrast to the 111 reflection, single patterns show a rich variety of maxima with respect to *l* that average out for the averaged correlation.

In Figs. 5 ▸(*c*) and 5 ▸(*d*) ensemble averages 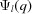 are shown for the crystalline and glassy samples, respectively. The crystalline sample exhibits strong contributions in the vicinity of the Bragg peaks only, which slightly drop with rising *l*. These results reflect the well developed orientational order in crystalline sample systems. In contrast, the glassy sample shows neither dominant peaks nor remarkable *q* dependence, except a decrease of all coefficients with increasing *q*. Furthermore, it has a significantly lower amplitude in the region of 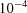 compared to that for the crystalline sample of 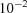
–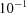. A more detailed discussion of the results for glassy samples will be given in a different publication (Lehmkühler *et al.*, 2017 ▸).

## 4. Summary and conclusion   

In summary, we have demonstrated the feasibility of using XCCA to access the structure of densely packed samples beyond the static structure factor 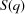. By calculation of intensity cross-correlations in the vicinity of Bragg reflections and comparison with model structures, we conclude that the studied crystal is of f.c.c. structure with indications of stacking faults, as expected for equilibrium hard-sphere colloidal crystals. In this way, the powder average can be overcome, allowing access to details of the structure of the crystal’s unit cell. In addition, we were able to track slight deviations from calculated correlations, most likely due to the size polydispersity of the particles, resulting in a weaker effective resolution. This is also expressed by the correlation between different Bragg reflections.

The XCCA method allows one to reveal higher-order structural correlations, for example crystalline materials showing a mixture of different crystal structures, and represents a new instrument for studies of densely packed systems. Furthermore, it will allow researchers to get access to structural correlation during phase transitions and provide insight into supercooled or glassy systems, where such higher-order correlations are suggested to be a fingerprint of ordered clusters or critical length scales (Steinhardt *et al.*, 1983 ▸; Tanaka, 2011 ▸; Leochmach & Tanaka, 2012 ▸). Thanks to the short and coherent pulses of FEL facilities in the hard X-ray regime (Gutt *et al.*, 2012 ▸; Lehmkühler *et al.*, 2014 ▸, 2015 ▸), correlation studies will reveal such processes extending to ultrafast time scales. By determining correlations between different *q* values and studying Fourier modes, further information can be obtained, for example, in multicrystalline systems to detect higher-order Bragg reflections or disentangle complex structures of both crystalline and amorphous materials.

## Figures and Tables

**Table 1:** *q* values and corresponding Bragg reflections of the f.c.c. and h.c.p. correlations studied in Fig. 3 ▸

	*q* (nm^−1^)	f.c.c. index	f.c.c. label	h.c.p. index	h.c.p label
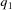	0.027	111	i	002	b
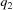	0.029	111/200	i and ii	011	c
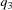	0.031	200	ii	–	–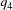	0.044	220	iii	110	e
